# What is the connection between screen time and lifestyle factors important for bone health? Findings from the Hertfordshire Intergenerational Study

**DOI:** 10.1007/s11657-026-01675-z

**Published:** 2026-02-19

**Authors:** Leo Westbury, Gregorio Bevilacqua, Faidra Laskou, Fiona Kirkham-Wilson, Nicholas Fuggle, Elaine Dennison

**Affiliations:** MRC Lifecourse Epidemiology Centre, University of Southampton, Southampton, UK

**Keywords:** Screen time, Lifestyle factors, Bone health

## Abstract

***Summary*:**

Screen time is increasing and may influence lifestyle factors relevant to bone health. In this study, more screen time was related to lower physical activity amongst males and individuals aged 21–52, and higher BMI amongst individuals aged 60–69. Greater screen time may increase the risk of poor bone health and obesity.

**Purpose:**

Screen time, whether through remote working or leisure pursuits, is rising in the adult population across all ages. Here, we consider how this relates to lifestyle factors important for bone health.

**Methods:**

Overall, 67 males and 136 females, aged 21–69, were analysed. BMI was derived from self-reported height and weight. Physical activity was ascertained using the International Physical Activity Questionnaire. Participants completed a food frequency questionnaire; a diet quality score was derived using principal component analysis. Screen time (past 7 days), smoking, alcohol intake, educational attainment, and comorbidities were self-reported. Screen time in relation to BMI, physical activity, diet quality, ever smoking, and high alcohol intake (> 14 units/week) was examined using linear and logistic regression as appropriate, with adjustment for education and number of comorbidities, and stratified by sex and age tertiles. Standard deviation (SD) differences in continuous outcomes (odds ratios for binary outcomes) per SD increase in screen time are reported.

**Results:**

Median (lower quartile, upper quartile) age was 56 (42, 60) years; corresponding statistics for screen time were 249 (152, 377) min/day. More screen time was related to lower physical activity, especially amongst males (−0.58 (95% CI −0.82, −0.35)) and those aged 21–52 (−0.47 (−0.71, −0.22)), higher BMI amongst those aged 60–69 (0.25 (0.03, 0.48)), and reduced odds of ever smoking amongst males (0.30 (0.10, 0.89)).

**Conclusion:**

These findings suggest that increased screen time is related to health behaviours which may increase the risk of poor bone health and obesity, with differing associations according to sex and age groups.

**Supplementary Information:**

The online version contains supplementary material available at 10.1007/s11657-026-01675-z.

## Background

Screen time, defined as the amount of time spent using devices with screens such as televisions, computers, tablets, and smartphones, has markedly increased since before the COVID-19 pandemic [1]. Reasons for this include the widespread adoption of remote working and shifts in routines that have reduced participation regarding in-person activities [2]. This includes increased screen time through social activities also linked to online activity. Together, these transitions have raised concerns about the potential health implications of prolonged screen time, particularly in relation to poor health behaviours such as increased sedentary behaviour, which may have serious health implications, including for the skeleton [2].

Whilst this topic has not been investigated in adults in midlife to our knowledge, many studies amongst children and adolescents have explored the potential impact of screen time on physical and mental health [3–5], with some reporting sex differences. For example, in Norwegian boys (aged 15–18 years) who participated in the Tromsø Study, time spent on screen-based sedentary activity was negatively associated with bone mineral density; this negative association was not present amongst girls [6]. However, research amongst adults examining screen time in relation to multiple health behaviours in the same cohort, such as physical activity, diet quality, smoking, and alcohol consumption, is limited. Moreover, it remains unclear whether these associations differ by sex or age, which may have implications for the design and targeting of public health interventions. Understanding how screen time relates to these health behaviours amongst older adults is important, as they are established determinants of both bone health and overall health [7].

Therefore, we examined associations between screen time and these health behaviours, along with BMI, in the Hertfordshire Intergenerational Study, a community-dwelling cohort of adults. Analyses were also stratified by sex and age to investigate whether associations differed according to these factors.

## Methods

### Study sample

The Hertfordshire Cohort Study (HCS) is a population-based study that was originally established to investigate the origins of adult diseases [8, 9]. It comprises community-dwelling individuals born in Hertfordshire, UK, between 1931 and 1939. Participants were recruited from 1998 to 2004, identified through birth records in Hertfordshire health visitor ledgers. Since their initial recruitment, HCS participants have engaged in multiple follow-ups, providing extensive information on their clinical, lifestyle, and sociodemographic characteristics.

The Hertfordshire Intergenerational Study was initiated in 2017, enrolling the original HCS participants’ children and grandchildren. Eligible descendants who were invited to participate were individuals aged 16 and older and residing in the UK. Of the 1090 postal questionnaires sent to the offspring of the original HCS participants, 746 were returned (462 children and 284 grandchildren). These 746 people were invited to attend a clinical visit in Cambridge, UK.

In 2022, the individuals in the Hertfordshire Intergenerational Study were invited to complete a follow-up questionnaire via post or email. This questionnaire collected information relevant for this analysis such as participants’ education, anthropometry, health behaviours and comorbidities. The window for completing the questionnaire ran from July to November 2022; participants were invited once to complete the questionnaire without the use of reminders, no incentives were offered, and the questionnaire was available only in English. Prior to distribution, the questionnaire was piloted amongst members of the Hertfordshire Cohort Study research team to assess clarity of the questions and estimated completion time. The Hertfordshire Research Ethics Committee granted ethical approval (REC reference: 16/LO/1225); all investigations were in accordance with the Declaration of Helsinki and its later amendments or comparable ethical standards; all individuals provided written informed consent.

### Participant information ascertained from the Hertfordshire Intergenerational Study follow-up questionnaire

All the information used in this current analysis was ascertained from this self-reported follow-up questionnaire. Participants were asked to report their height and weight. Information was provided in the questionnaire on the typical number of units in different types of alcoholic drinks to enable participants to report their alcohol consumption; smoking status (never smoker, ex-smoker or current smoker) was also ascertained. To ascertain screen time, participants were asked how long they spent watching TV or looking at a screen over the past 7 days. Participants were asked to report how many days they worked from home each week. Physical activity was assessed using the International Physical Activity Questionnaire (Short Form) [10]. Participants reported the frequency (number of days per week) and duration (minutes per day) of activities performed for at least 10 minutes at a time during the previous 7 days, across four domains: vigorous-intensity activity, moderate-intensity activity, walking and sitting. A food frequency questionnaire was used to calculate a prudent diet score for each participant to reflect their diet quality [11]; higher scores reflected healthier diets. Highest level of education (GCSE or equivalent, A-level or equivalent, university degree or equivalent, postgraduate degree, and other) was also ascertained. Participants were asked to indicate whether a doctor had ever told them that they had experienced the following conditions: heart attack or angina; stroke or transient ischaemic attack; high blood pressure; high cholesterol; type 2 diabetes; asthma, bronchitis, emphysema or COPD; cancer; depression or anxiety; stomach or digestive problems; osteoporosis; rheumatoid arthritis; and osteoarthritis.

### Derivation of variables prior to analysis

Self-reported weight and height were used to derive BMI. Smoking history was dichotomised into never smoked and ever smoked. Participants reporting more than 14 units of alcohol per week were classed as having a high alcohol intake. Total physical activity for each category (vigorous-intensity activity, moderate-intensity activity, walking, and sitting) was calculated by multiplying the weekly duration of each activity category in minutes by its corresponding metabolic equivalent (MET) value. The resulting MET-minutes per week for each category were summed to derive total physical activity. Educational attainment was grouped into four categories: GCSE or below, A-level or equivalent, university degree or equivalent, and postgraduate degree. The number of comorbidities, out of the list of doctor-diagnosed conditions, was calculated and used as a marker of morbidity level.

### Statistical methods

Participant characteristics were characterised using descriptive statistics such as means, standard deviations, medians and interquartile ranges, and frequency and percentage distributions. Screen time in relation to physical activity, diet quality, BMI, smoking (ever versus never) and alcohol consumption (> 14 units/week vs less) was examined using linear and logistic regression, as appropriate, with adjustment for sex, age, educational attainment and number of comorbidities. Analyses were stratified first by sex and then by age tertiles; all stratified analyses were adjusted for age, educational attainment and number of comorbidities, and the age-stratified analyses were also adjusted for sex. Continuous exposures and outcomes were standardised so that they had a mean of zero and a standard deviation of one to aid comparison of effect sizes. Analyses were conducted using R, version 4.4.1.

## Results

### Descriptive statistics

In total, 307/746 (41%) participants completed the 2022 questionnaire; the analysis sample comprised the 203/307 participants (67 males and 136 females) with complete data on the exposures, outcomes and adjustments used in the analysis. Table 1 shows the participant characteristics of the analysis sample. Median (lower quartile, upper quartile) age was 55 (36, 60) years amongst males and 57 (50, 60) amongst females; corresponding statistics for screen time were 300 (180, 424) min/day amongst males and 240 (137, 360) amongst females.

**Table 1:** Participant characteristics of the analysis sample

Participant characteristic	Mean (SD); median (lower quartile, upper quartile); *n* (%)	
	Males (***n*** = 67)	Females (***n*** = 136)
Age (years)	55 (36, 60)	57 (50, 60)
BMI (kg/m^2^)	25.4 (3.9)	25.6 (5.6)
Ever smoked	10 (14.9%)	28 (20.6%)
High alcohol intake (> 14 units/week)	17 (25.4%)	22 (16.2%)
Diet quality score	1.6 (1.3)	2.1 (1.3)
Physical activity (MET-minutes/week)	2754.0 (1540.5, 5283.0)	2039.0 (1354.1, 3534.8)
Screen time (mins/day)	300 (180, 424)	240 (137, 360)
Home working (days/week)*	3 (0, 4)	2 (0, 4)
Educational attainment		
GCSE or below	6 (9.0%)	17 (12.5%)
A-level or equivalent	12 (17.9%)	34 (25.0%)
University degree or equivalent	28 (41.8%)	50 (36.8%)
Postgraduate degree	21 (31.3%)	35 (25.7%)
Number of comorbidities	0 (0, 1)	1 (0, 2)

^*^Amongst those who were working full-time, part-time or self-employed

Descriptive statistics regarding age, sex, current smoking and alcohol intake at the baseline stage of the Hertfordshire Intergenerational Study (*n* = 746) are presented in Supplementary Table 1, stratified according to whether or not participants were included in the analysis sample (*n* = 203). Compared to participants who were not included in the analysis sample, those who were included were generally older, but other participant characteristics were similar between the two groups (*p* > 0.05 for the other participant characteristics).

### Associations between screen time and other health behaviours

Standard deviation (SD) differences in physical activity and diet quality, and odds ratios for ever smoking and high alcohol intake, are shown in Table 2 for the whole analysis sample per SD increase in screen time. More screen time was related to lower physical activity after adjustment for sex, age, educational attainment and number of comorbidities (−0.35 (95% CI −0.48, −0.21), *p* < 0.001). Associations regarding screen time in relation to BMI and the other health behaviours were weak (*p* > 0.25 for all associations). Table 3 presents associations between screen time and the outcomes after stratification by sex and age tertiles. More screen time was related to lower physical activity amongst males (−0.58 (−0.82, −0.35), *p* < 0.001) and amongst those aged 21–52 (−0.47 (−0.71, −0.22), *p* < 0.001). More screen time was also related to greater BMI amongst those aged 60–69 (0.25 (0.03, 0.48), *p* = 0.027) and reduced odds of ever smoking amongst males (0.30 (0.10, 0.89), *p* = 0.030). None of the stratified associations regarding screen time in relation to diet quality and alcohol consumption were statistically significant (*p* > 0.08 for all associations).

**Table 2:** Standard deviation difference in outcomes per standard deviation increase in screen time after adjustment for sex, age, educational attainment and number of comorbidities

Outcome	Estimate (95% CI)	*p*-value
Physical activity (*z*-score)	− 0.35 (− 0.48, − 0.21)	< 0.001
Diet quality (*z*-score)	− 0.08 (− 0.22, 0.06)	0.258
BMI (*z*-score)	0.06 (− 0.08, 0.20)	0.380
Ever smoking (odds ratio)	1.00 (0.68, 1.47)	0.989
High alcohol intake (odds ratio)	0.87 (0.59, 1.26)	0.453

High alcohol intake: > 14 units/week

*SD* standard deviation

**Table 3:** Standard deviation difference in outcomes per standard deviation increase in screen time, stratified by sex and age

Outcome	Group	Estimate (95% CI)	*p*-value
Physical activity (*z*-score)	Males only	− 0.58 (− 0.82, − 0.35)	< 0.001
	Females only	− 0.17 (− 0.34, 0.00)	0.052
	Lowest age tertile (21–52 years)	− 0.47 (− 0.71, − 0.22)	< 0.001
	Middle age tertile (53–59 years)	− 0.20 (− 0.41, 0.00)	0.056
	Highest age tertile (60–69 years)	− 0.24 (− 0.49, 0.01)	0.067
Diet quality (*z*-score)	Males only	− 0.16 (− 0.38, 0.06)	0.150
	Females only	− 0.05 (− 0.23, 0.14)	0.627
	Lowest age tertile (21–52 years)	− 0.02 (− 0.30, 0.27)	0.913
	Middle age tertile (53–59 years)	− 0.16 (− 0.37, 0.04)	0.114
	Highest age tertile (60–69 years)	− 0.05 (− 0.32, 0.22)	0.708
BMI (*z*-score)	Males only	0.15 (− 0.03, 0.33)	0.100
	Females only	− 0.01 (− 0.21, 0.19)	0.931
	Lowest age tertile (21–52 years)	− 0.10 (− 0.36, 0.15)	0.414
	Middle age tertile (53–59 years)	− 0.01 (− 0.28, 0.26)	0.943
	Highest age tertile (60–69 years)	0.25 (0.03, 0.48)	0.027
Ever smoking (odds ratio)	Males only	0.30 (0.10, 0.89)	0.030
	Females only	1.24 (0.76, 2.01)	0.385
	Lowest age tertile (21–52 years)	0.71 (0.31, 1.62)	0.416
	Middle age tertile (53–59 years)	0.91 (0.44, 1.91)	0.811
	Highest age tertile (60–69 years)	1.42 (0.72, 2.80)	0.307
High alcohol intake (odds ratio)	Males only	0.69 (0.37, 1.29)	0.244
	Females only	1.04 (0.63, 1.74)	0.868
	Lowest age tertile (21–52 years)	0.75 (0.38, 1.48)	0.404
	Middle age tertile (53–59 years)	1.86 (0.91, 3.82)	0.089
	Highest age tertile (60–69 years)	0.50 (0.21, 1.16)	0.107

All associations were adjusted for age as a continuous variable, educational attainment and number of comorbidities; associations within each age tertile were also adjusted for sex. High alcohol intake: > 14 units/week

*SD* standard deviation

## Discussion

This study of community-dwelling adults, with a median (lower quartile, upper quartile) age of 56 (42, 60) years, found that higher levels of screen time were associated with lower physical activity, especially amongst males and amongst those aged 21–52 years. Additionally, greater screen time was related to higher BMI in older adults aged 60–69 years and lower risk of ever smoking amongst males. These findings suggest that screen time is related to a range of health behaviours, with patterns varying by age and sex. These observations are relevant as weight bearing physical activity, adiposity and smoking behaviours are all associated with bone health [7]. Because educational attainment may be associated with a higher likelihood of home working, analyses were adjusted for this variable.

Previous studies have examined screen time in relation to health behaviours amongst adults in other countries, largely based in Asia and America. In a study comprising 7808 Korean adults, aged 19 to 69 years, participants with over 6 h/day of screen time had 1.42 (95% CI 1.08, 1.86) times greater odds of obesity compared to those with less than 2 h/day of screen time [12]. Similarly, a Japanese study, comprising 2488 participants (aged 20 years and older), reported associations between increased screen time and greater risk of obesity amongst all participants, with larger effect sizes amongst those aged at least 65 years [13]; these latter results support our observation of stronger associations between greater screen time and higher BMI amongst those aged 60–69 years. In a US study comprising 926 adults, aged 18 years and older, the heaviest screen time users reported the least healthy dietary patterns (few fruits/vegetables and regularly consumed sweet drinks), the highest frequency of fast-food consumption and the lowest amount of physical activity [14]. Whilst our study found associations between more screen time and lower physical activity, associations regarding diet quality were weak. In a Brazilian study comprising 1897 adults with a mean (SD) age of 37.9 (13.3) years, increased television and cell phone use were associated with increased sweetened food consumption during the COVID-19 pandemic, whilst increased computer time was a protective factor in these relationships; weak associations were observed regarding smoking [15]. Further evidence of relationships differing according to the type of screen time, albeit in adolescents, was reported in a cross-sectional study in Brazil comprising 845 participants with a mean (SD) age of 16.4 (1.1) years [16]. In this investigation, increased study-related screen time was associated with a lower risk of smoking and consuming alcohol in the past 30 days, whereas increases in social media use were associated with increased risk of these outcomes. Another study amongst 1038 adolescents of the Tromsø Study, aged 15–18 years, reported differing associations by sex; screen time at weekends was positively related to BMI and negatively associated with bone mineral density amongst boys, whereas increased screen time at weekends amongst girls was related to greater bone mineral density, with much weaker associations than boys regarding BMI [6]. The apparent sexual dimorphism is interesting and may reflect different types of screen time; possibly men may be more likely to work full-time in a largely sedentary role, whilst women may combine part-time remote working with more smart phone use.

This study has several strengths. It considered a broad range of health behaviours, including physical activity, diet quality, smoking status, alcohol consumption, as well as BMI, providing a comprehensive examination of how screen time may relate to multiple aspects of lifestyle. The inclusion of participants across a wide age range enabled exploration of whether associations varied by age group. Although only 27% (203/746) of the original Hertfordshire Intergenerational Study participants were included in the analysis sample, distributions of sex, smoking status and alcohol consumption at the baseline Hertfordshire Intergenerational Study were similar between the analysis sample and the group (*n* = 543) who were not included in the analysis sample. However, this study also has limitations. First, the sample size was small (*n* = 203), which would have limited the statistical power to detect associations. For example, assuming a two-sided significance level of *p* < 0.05, 80% power and a standardised predictor and outcome used in a simple linear regression model, this sample size results in a minimum detectable effect size of 0.19 (0.19 standard deviation difference in outcome per one standard deviation increase in predictor); this minimum detectable effect would be even greater in the stratified analyses. Furthermore, when analyses were stratified by age, some strata encompassed a wide age range. Second, all health behaviours, including screen time, were self-reported, which may have introduced social desirability or recall bias. Third, we did not collect information on the amount of screen time that was due to work or leisure, or the time spent on different types of electronic devices. Fourth, participants completed the questionnaires between July and November 2022, only a few months after COVID-19 legal restrictions were lifted in England (February 2022) [17]. As a result, pandemic-related changes, such as increased remote working and remote learning, may still have been influencing screen time behaviours and other health behaviours at the time of data collection. Consequently, the generalisability of these findings to periods several years after the COVID-19 pandemic may be limited. Fifth, only cross-sectional associations were examined, limiting the ability to infer causality from the associations reported. Six, use of the short-form International Physical Activity Questionnaire has limitations. It relies on self-reported data, which often leads to overestimation of physical activity compared with objective measures, showing only weak-to-moderate correlations with objective assessments in previous studies [18]. Participants may also find it difficult to distinguish between moderate- and vigorous-intensity activities, potentially resulting in inaccurate total activity estimates. Furthermore, this questionnaire does not capture details about activity types such as weight-bearing, resistance or high-impact exercise, factors particularly relevant to bone health [19]. Finally, whilst screen time was examined in relation to a range of health behaviours that are known to be important for bone health, direct analysis of its association with bone health was not possible, as bone parameters were not measured in this study. Menopausal status, an established influence on bone mineral density and bone health in general [20], was also unavailable.

The findings of this study highlight the complex interplay between screen time and health behaviours across different demographic groups, with potential implications for the targeting of health intervention strategies. The association between more screen time and lower physical activity, particularly amongst males and younger adults, underscores the need for interventions that address sedentary screen-based behaviours as a barrier to active lifestyles. For example, incorporating digital prompts or app-based nudges to encourage breaks from screen time could help counteract prolonged periods of sedentary behaviour amongst heavy screen users. The association between more screen time and higher BMI amongst older adults suggests that this group may be particularly vulnerable to the cumulative effects of prolonged sedentary behaviour, reinforcing the importance of support around healthy screen use in this group. Interestingly, more screen time was linked to reduced odds of ever smoking amongst males, which may reflect shifts in social norms or displacement of time that would otherwise be spent in smoking-related activities. These results suggest that screen time may be considered not only as a risk factor for physical inactivity but also as a potential influence on broader health behaviours. Interventions could be devised to reflect the different associations observed within various groups, although the associations identified in this cohort require replication in larger studies.

Future research could expand on these findings by using larger cohorts with objectively measured physical activity data from accelerometers or similar devices to accurately capture total physical activity levels. Furthermore, more detailed information on the type of activities performed, including weight-bearing activities, and a breakdown of screen time according to device type and purpose (work, leisure or education), would be valuable. In addition, incorporating this information with participant data on bone mineral density and fracture history could enhance understanding of how specific activity patterns influence bone health and support the development of targeted health prevention strategies.

In conclusion, except for a lower likelihood of ever smoking amongst males, higher screen time was associated with less favourable patterns of physical activity and BMI in various groups of participants. These findings suggest that increased screen time may have broader negative implications for adult health, including potential adverse effects on bone health.

## Supplementary Information

Below is the link to the electronic supplementary material.

ESM 1 Supplementary Material 1 (DOCX 16.3 KB)

## Data Availability

The data used in this article cannot be shared widely due to consent restrictions. The participants only consented for their data to be shared with the Hertfordshire Cohort Study Research Team. Requests to access Hertfordshire Cohort Study data for new research projects should be made to ED (emd@mrc.soton.ac.uk).
