# Supplementary material for: What is the connection between screen time and lifestyle factors important for bone health? Findings from the Hertfordshire Intergenerational Study
Source: Arch Osteoporos. 2026 Feb 19;21(1):40. doi: 10.1007/s11657-026-01675-z (PMC12917093; doi:10.1007/s11657-026-01675-z)
Supplement: Supplementary file 1 — Supplementary Material 1 (DOCX 16.3 KB) [file 11657_2026_1675_MOESM1_ESM.docx]

| **Supplementary Table 1: Participant characteristics at the baseline Hertfordshire Intergenerational Study, stratified according to whether or not participants were included in the analysis sample** | | |
| --- | --- | --- |
|  |  |  |
|  |  |  |
| **Participant characteristic** | **Mean (SD); median (lower quartile, upper quartile); or n(%)** | |
|  | **Analysis sample**  **(n=203)** | **Not included in analysis sample (n=** **543)** |
| Age (years) | 52 (37, 56) | 48 (26, 55) |
| Sex (female) | 136 (67.0%) | 330 (60.8%) |
| Current smoker | 5 (2.5%) | 31 (5.7%) |
| Alcohol intake (units/week) | 6.6 (1.2, 12.8) | 5.7 (0.8, 13.4) |

*p<0.001 for differences in age between the two groups; for all other participant characteristics, differences between the two groups were not statistically significant (p>0.05)*
